# Application of K-means algorithm to Werner deconvolution solutions for depth and image estimations

**DOI:** 10.1016/j.heliyon.2022.e11665

**Published:** 2022-11-17

**Authors:** Daniel Eshimiakhe, Kola Lawal

**Affiliations:** Department of Physics, Ahmadu Bello University, Nigeria

**Keywords:** Clustering algorithm, Potential field, Werner deconvolution

## Abstract

One of the most popular techniques for computer-assisted solution estimates for magnetics and gravity field data is Werner deconvolution. The approaches frequently produce erratic results and may not always forecast the maximum number of the geologic entity that produces them due to the intrinsic instability of potential field data. This led to the application of the K-means machine learning algorithm to further enhance the detection of the geologic potential field-generated bodies. Two substances that resembled dikes were combined to form a synthetic magnetic model. Random noise was added to the synthetic data, to make the solutions a bit more complex. Werner deconvolution technique was applied to the synthetic model to generate solutions. K-means unsupervised machine learning algorithm was applied to the generated solutions created by the synthetic data. We further applied this algorithm to real data sets from a mining site. The clustering result shows a good spatial correspondence with the geologic model, and the method was able to estimate the precise location and depth of the dike bodies. The proposed method is entirely data-driven and has proven to work in the presence of noise.

## 1 Introduction

The Earth System is subject to a variety of geohazards that are not properly understood, but they are having significant impacts as our exposure and insight continue to increase through increased urbanization. Mapping of geological features is an area of research made possible by the development of large-scale, high-resolution geophysical data collection, and one of these geophysical techniques is the potential field. The objective of the magnetic survey is to investigate anomalous geological features, that may alter the observed magnetic field. Because the magnetic properties of the underlying rocks are different, there are fluctuations in the magnetic field. Numerous magnetic interpretation techniques have been created to ascertain the depth of diverse geometric shapes' geological structures.

The strategies are based on: (i) graphical methods utilizing some distinctive features of the magnetic profile (Koulomzine et al., 1970; Am, 1972; Subrahmanyam and Prakasa Rao, 2009); (ii) spectral analysis techniques (Prakasa Rao et al., 1986); and (iii) numerical techniques such as the Werner Deconvolution method (Hartman et al., 1971; Ku & Sharp; McGrath and Hood, 1973; Silva, 1989; Dondurur and Pamukcu, 2003). Large potential field data necessitate automatic interpretation techniques like Euler's algorithm and Werner deconvolution (Thompson, 1982; Werner, 1953). Werner created a technique for analyzing the magnetic anomalies of two-dimensional sheets-like structures that arranges the anomalies and separations from observation points along a profile to create a linear equation with coefficients related to the sheet's characteristics.

From the perspective of mathematical physics, the inversion of potential field data in geophysics belongs to ill-posed problems. The ambiguity of the solution position results from the inherent instability of potential field data inversion. Numerous solutions to this arrogant conundrum have been offered over the past few decades (Blakely, 1995; Mudretsova, 1990). This approach is based on the estimation of the position of the source under the assumption of a simple anomalous body. The so-called deconvolution or semi-automated method category includes this strategy. As an inverse procedure to the direct issue in potential fields theory, which is characterized by a convolution integral, Hartman et al. (1971) coined the term "deconvolution." The deconvolution procedure for a sheet in magnetometry was described by Kilty in 1983. We are emphasizing this information so that you won't be misled by the deconvolution process utilized in processing seismic data.

The starting values of the parameters are not necessary for Werner's technique. Based on clusters of fake positions at the locations of sheets, there has been an emerging trend to use the Werner deconvolution method to trace foundation structures (Ku and Sharp, 1983; Malleswara Rao et al., 1983; Thakur et al., 2000). To discover the best answers, Thompson (1982) presented clustering, which tends to group into so-called clusters (grouped arrangements of solutions). However, the number of geologic entities forming such clusters is unknown. The main goal of our contribution is to study the properties of Werner deconvolution and apply the K-means unsupervised machine learning algorithm to introduce an efficient clustering algorithm to detect with precision the exact location and number of the geologic body that generate such solutions, as one can often not determine how many geological bodies are clustered from a potential field data when Werner deconvolution is carried out.

Three factors limit the accuracy and utility of the Werner deconvolution method in practice. First, analysis is limited to simple 2-D models such as dipping dikes, interfaces, and horizontal cylinders, with a minimum resolution criterion between neighboring bodies. Because Hartmann et al. (1971), Jain (1976), and Godson (1978) addressed these constraints. They are not the focus of this paper. Second, the matrix of coefficients involved in the system of equations solution is generally-conditioned, making its inversion very susceptible to noise; this is why the K-means machine algorithm was used to solve the problem.

According to Radhakrishna Murty et al. (2000), clusters are only restricted to contacts and faults in basements when they are far apart and extremely steep. Werner's technique does not offer a trustworthy interpretation for structures with smooth edges, modest dipping, proximity, and moderate depth. Although the source of this study is a vertical thin dike, it can also be used for other types of bodies if it is assumed that the body is made up of multiple thin dikes. The procedure is easy, quick, and trustworthy when it is limited to interpreting the anomalies of isolated simple geometric bodies. These methods' depth estimations could be used as rough starting points for modeling techniques.

Machine learning algorithms have been adopted in geophysical studies, such as exploration geophysics, earthquake localization, aftershock pattern analysis, and earth system analysis (Huang et al.*,* 2017; Helmy et al.*,* 2010; Jia and Ma, 2017; Lim, 2005; Poulton, 2002; Zhang et al.*,* 2014, Mousavi et al.*,* 2016, DeVries et al., 2018, Reichstein et al., 2019). This paper focuses on the application of the K-means clustering algorithm to Werner deconvolution solutions to predict optimal results from solutions generated by Werner deconvolution. This method looks at the possibility of detecting the specific number of geologic bodies that generate the solutions generated by Werner deconvolution.

## 2 Methodology

We grasp the profile technique under the 2D interpretation method based on the notion of a 2D body. An interpretation equation is converted into a set of linear algebraic equations, and the unknown parameters are then solved. The n-point window traveling along the profile is used to determine the input parameters. The parameters of the specified body type are generated from the linear system solution vector. In the following, we'll refer to the spatial position of the computed result as the solution/source position. One method is based on Werner deconvolution and the other is the K-means machine algorithm. The Werner deconvolution method was applied to magnetic anomalies over dike models. The synthetic magnetic data were generated for the tabular model by varying the half-width to depth (w/H) ratio for the dike.

A geological model was first created by generating synthetic magnetic data to test and prove the effectiveness of the K-means unsupervised machine learning algorithm in determining clusters around an anomalous body. The parameters of the model consisted of two bodies (dike-like in form) located at a fixed depth. Werner deconvolution technique was applied to generate the magnetic solutions as clusters. The bodies were dike-like structures in shape and the solutions were plotted.

The most commonly used unsupervised clustering algorithms make use of partitioning the data into a predeﬁned number of clusters (Witten et al., 2017). The k-means (orc-means) algorithm separates data into k clusters which are deﬁned by randomly assigning centers. The distance between a sample and a randomly assigned center is calculated and the sample is assigned to the closest center. The algorithm then calculates a new center to the deﬁned cluster and iterates through the same process until the algorithm converges to a minimum (Marsland, 2009).

### 2.1 Werner deconvolution

Werner (1953) suggested a method for its interpretation after analyzing the magnetic field of random dikes. By solving the systems of linear equations, we can determine the top corners of an object either assuming a thin aisle from the T-curve or assuming a thick aisle from the (T)/x-curve. The approach does offer a wide range of applications, despite the simplifications appearing to be a constraint.

(Hartman et al., 1971; Jain, 1976; Kilty, 1983; Ku and Sharp, 1983; Klingel'e et al., 1991) conducted additional research on Werner deconvolution. Consider a brief overview of the "Werner" n-point operator's characteristics. When we use an n-point operator, we are solving n equations with n or fewer unknowns.

Let us write the interpretation equation for an arbitrary spatial position of the dike (x_0_,h_0_) on a proﬁle (x, 0) with interference polynomial coeﬃcients $c_{i}$ of degreeincrement.

(1) $$\Delta T\left(x,0\right)=\frac{A\left(x-x_{0}\right)+Bh_{0}}{{\left(x-x_{0}\right)}^{2}+h_{0}^{2}}+c_{0}+c_{1}x+c_{2}x^{2}+---+c_{m}x^{m}$$

Or

(2) $$-b_{0}T-b_{1}Tx+Tx^{2}=a_{0\;}+a_{1}x+a_{2}x^{2}+---+a_{m}x^{m+2}$$

Where;

$$b_{0}=-x_{0}^{2}-h_{0}^{2}$$

$$b_{1}=2x_{0}$$

$$a_{0}=\left(Bz_{0}-Ax_{0}\right)+c_{0}\left(x_{0}^{2}+h_{0}^{2}\right)$$

$$a_{1}=A-2c_{0}x_{0}+c_{1}\left(x_{0}^{2}+h_{0}^{2}\right)$$

(3) $$a_{2}=c_{0}-2c_{1}x_{0}+c_{2}\left(x_{0}^{2}+h_{0}^{2}\right)$$

$$a_{k}=c_{k-2}-2c_{k-1}x_{0}+c_{k}\left(x_{0}^{2}+h_{0}^{2}\right)$$

$$a_{n}=c_{n-2}$$

From Eq. (2) we have:

$$x_{0}=b_{1}/2$$

$$z_{0}=\sqrt{{-b}_{0}-b_{1}^{2}}/4$$

$$d_{n}=a_{n}$$

$$d_{n-1}=a_{n-1}+2d_{n}x_{0}$$

(4) $$d_{n-2}=a_{n-2}-2d_{n-1}x_{0}-d_{n}\left(x_{0}^{2}-h_{0}^{2}\right)$$

$$A=d_{1}=a_{1}-2d_{2}x_{0}-d_{3}\left(x_{0}^{2}-h_{0}^{2}\right)$$

B=d0h0=[a0−d1x0−d2(x02−h02)]h0

Where $d=d_{0},d_{1},d_{2},-----d_{n}$ is “solution” vector, n+1 is the number of terms on the right-hand side of Eq. (2). The number of terms on the right-hand side of the equation is n+1, and the "solution" vector is given by the notation d = d 0,d 1,d 2,—-d n (2). From parameters A and B, we can compute dip and susceptibility as well as the parameters of the interference polynomial 0, 1, and 22. Eq. (4) provides the solution for the depth and location of the dike.

From parameters A and B, we can compute dip and susceptibility, and parameters of the interference $c_{0},c_{1},c_{2},-----c_{n-2}$.

As long as we have computed parameters A, B we can derive susceptibility contrast and the dip of the dike.

(5) $$k=\frac{M_{l}^{2}+M_{t}^{2}}{\left|T\right|}$$

(6) $$\emptyset =\arctan\frac{M_{t}}{M_{l}}+I$$

where I is magnetic inclination of the inducing ﬁeld T.

We now try to investigate how the estimated parameters affect the solution, taking into account all the equations. All relevant data regarding the dike's magnetic inclination, declination, strike, dip, and susceptibility (parameters $a_{i}$) are contained on the right-hand side of Eq. (2). It is evident that modifications to the dike's $a_{i}$ parameters, which are related to the right-hand side of Eq. (2), have no effect on the depth and location of a dike for the supplied data. Rarely are we able to determine the precise location of a geological body without knowing any other details about it. On the other hand, as shown in equations, the parameters $b_{i}$ have an impact on the tilt, strike, and magnetization solutions (3).

### 2.2 K-means clustering

Our computational strategy is to compute the results for all meaningful ranges of the input parameters (window width, interference polynomial, structural index).

K-means cluster analysis is an example of a hard partitioning algorithm (Hartigan, 1975; Hartigan and Wong, 1979; Jain et al., 1999). Consider a set of P data $\left(x_{1}\;,x_{2}\;,----,x_{P}\right)$ in 'd' dimensions which are partitioned into K clusters, where each element in the dataset is allocated entirely to a particular cluster.

The chance of recognizing regularities and latent patterns inside any piece of data is what we are interested in whenever we want to cluster it. In practice, we must select how we wish to characterize and search for structures inside a data. The most popular answers to these questions are given by the K-means machine learning algorithm. Structures are represented as subsets of X, and the data is divided into a specified number (k) of clusters. In machine learning, K-means clustering is a fundamental and well-understood technique for splitting data into clusters represented by centroids.

(7) $$X=x_{1},x_{2},x_{3},----x_{\rho }$$

(8) $$S=\left\{S_{1},S_{2},----S_{\rho }\right\}$$

Where;

(9) $$S_{j}=\left\{\;S_{1},S_{2},----S_{D}\right\}$$

By minimizing the objective function

(10) $$J=\sum_{i=1}^{\rho }\;\sum_{j=1}^{k}{\left(\|x_{i}-S_{j}\|\right)}^{2}$$

The type of optimization used to minimize *J* is specific to the k-means cluster implementation.

As a result of reducing Eq. (8), suitable centroids S_j_ are determined so that the distances between data points and their closest cluster centroid are as small as possible when the data is partitioned into distinct clusters C.

In a word, the k-means method reduces Eq. (8) to its simplest form by using a greedy iterative update technique. It randomly initializes the parameters $S_{1}^{\left(t\right)},S_{2}^{\left(t\right)},---S_{k}^{\left(t\right)}$. When it is launched, say at iteration t = 0. The data is then clustered according to this initial guess. This simply means that the program finds k groups in which;

(11) $$C_{i}=\{x_{j}\;\varepsilon \;X|{\left|\left|x_{i}-S_{j}^{\left(t\right)}\right|\right|}^{2}\leq {\left|\left|x_{i}-S_{j}^{\left(t\right)}\right|\right|}^{2}\forall I\neq j$$

After clusters have been identified, the algorithm computes to update the current estimations of cluster centroids.

(12) $$S_{j}^{\left(t+1\right)}=\frac{1}{n_{j}}\sum_{x_{j}}x_{j}$$

The number of items in cluster $C_{i}$ is denoted by $n_{i}=\left|C_{i}^{\left(t\right)}\right|$. The iteration counter is increased to t = t + 1 and steps (8) and (9) are repeated until the assignment of data points to each cluster does not change any longer or t exceeds a predefined number of iterations.

It can be shown that updating each centroid to the sample mean of its cluster will not increase the value of objectives in Eq. (5) or (7). Therefore, each iteration improves on the previous result and k-means clustering usually converges quickly. However, as the algorithm starts from a random initialization, it cannot be guaranteed to ﬁnd the minimum of its objective function. Running k-means a few times to empirically determine appropriate clusters is critical, as it often converges to a local minimum.

Once the selected Werner solution data set has been divided into coherent and undesirable solutions, the centers x,y, and z of the c clusters within the filtered data set are obtained using the K-means algorithm. The primary geologic structures being mapped are represented by these groupings. The algorithm's primary flaw is that the number of clusters c must be determined before performing the computation (Balasko et al., 2005). Another option is to perform the clustering for various c values before computing validity indices for each division to evaluate the merits of each.

### 2.3 Determination of geologic strike of sources mapped

The interpreter must provide a distance threshold around each cluster so that it only tracks points that are close to it. Currently, all clusters share a single threshold. It is possible to set a threshold for each cluster separately, but this would necessitate more human intervention and reduce automation somewhat. The value of this common threshold was determined in our case by using cluster centers.

## 3 Synthetic magnetic model

**Figure 1:**
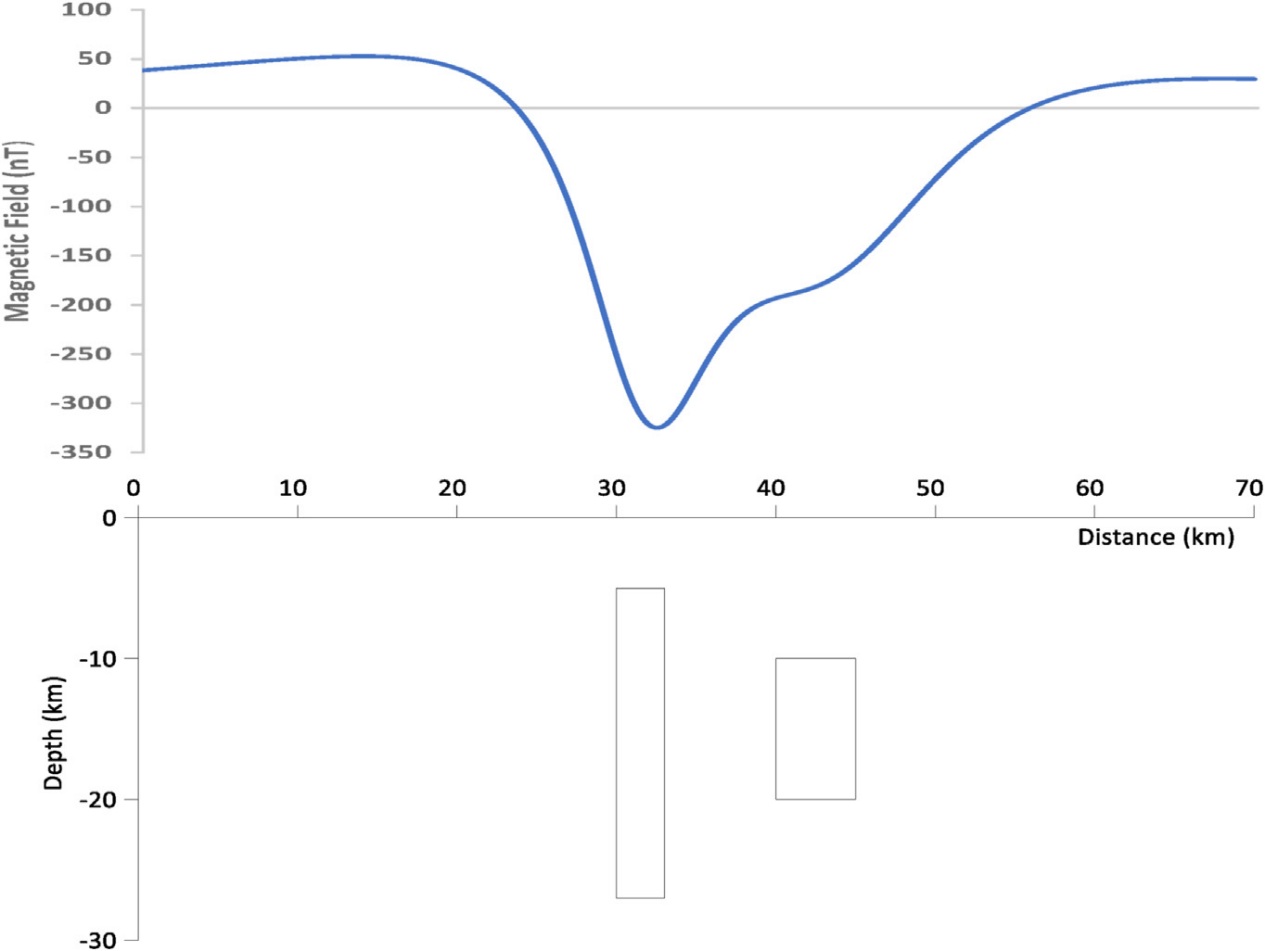
Magnetic field intensity produced by the two bodies (A and B) with field intensity of 33000 nT and magnetic inclination and declination of 4°and86° respectively.

The efficiency of this technique is investigated by generating synthetic data by using the method of Barse (Barse et al., 2003) to generate synthetic data. Both simulated and actual data were used to evaluate the algorithm. The synthetic model comprised of two substantially elongated bodies to test the algorithm's capacity to solve for the strike of geologic structures, which was one of the key objectives (Figure 1 and Table 1). Both bodies were 30 m deep and 3 m thick. The east-west body was 45 km long while the north-south body was 30 km long. In both instances, the depth to the top was 50 m. The 'north-south body' and 'east-west body' dip at 45° to the east and south, respectively. The ambient field's characteristics were declination 0°, inclination 90°, and total intensity 33,000 nT. There was no extra remanent magnetization. Both bodies' structural magnetic susceptibilities were 0.0035. At a common observation level of 0 m and with north-south lines separated at 500 m, a total magnetic intensity (TMI) grid was generated. The lines were spaced 10 m apart. A common minimal curvature approach was used to grid the forward model at a 100-m grid cell size after it was derived on profiles.

**Table:** 1Model parameters assuming an ambient field of 33,000 nT with 4° and 86° as magnetic inclination and declination respectively.

Body	Width (m)	Depth to top (m)	Depth to bottom (m)	Horizontal position (at midpoint) (m)	Susceptibility (SI unit)
A	3.0	5.0	27.0	31.5	0.01
B	5.0	10.0	20.0	42.5	0.01

Werner deconvolution applied on ΔT ﬁeld of two thin dyke with parameters: inducing ﬁeld: T = 33000 nT, inclination = 4^◦^, declination = 86^◦^. The total field parameter was chosen for this synthetic model based on the total residual magnetic field in Nigeria, and the inclination and declination were gotten.

**Figure 2:**
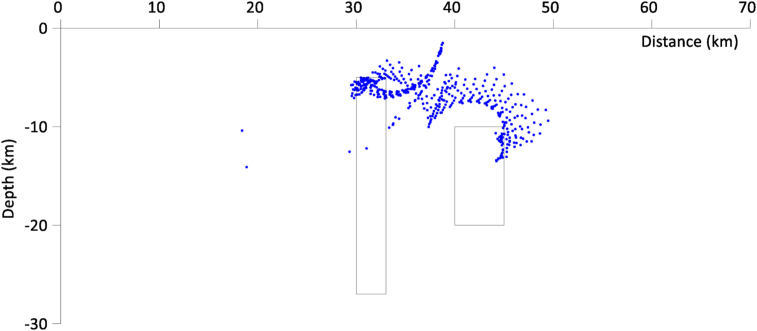
Magnetic solutions generated by the two geologic structures (the blue color represents the solutions). with field intensity of 33000 nT and magnetic inclination and declination of 4°and86° respectively.

The Werner deconvolution technique was applied to the residual field and the result is shown in Figure 2. Only solutions for dyke model were plotted since the bodies are dyke-like in shape. As can be seen, many solutions clustered around the depth to the top of the bodies but it would still have been difficult to ascertain the number of bodies in the model. To concentrate the solutions near the top of the bodies, a window of 100 × 100 m was used. The spray effect observed in the depth solutions is what is usually encountered when dealing with noise-free data. Random noise was therefore added to the data to make it more realistic. This noise was created by adding normally distributed random values to the theoretical magnetic anomaly of the dike body.

The theoretical magnetic anomaly of a single dipping dike was added with normally distributed random values to examine the impact of noise on the deconvolution process. The location and depth of the dike were then calculated using Werner deconvolution.

**Figure 3:**
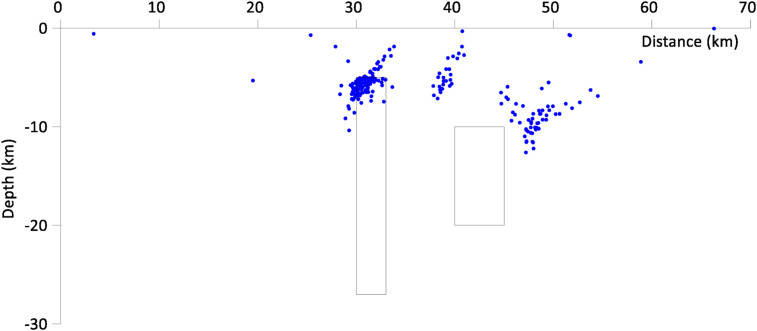
Magnetic solutions generated by the two geologic structures with random noise added (the blue color represents the solutions).

The result of this shown in Figure 3, even though devoid of sprays indicates (based on the clustering observed) that there are most likely three bodies in the model.

**Figure 4:**
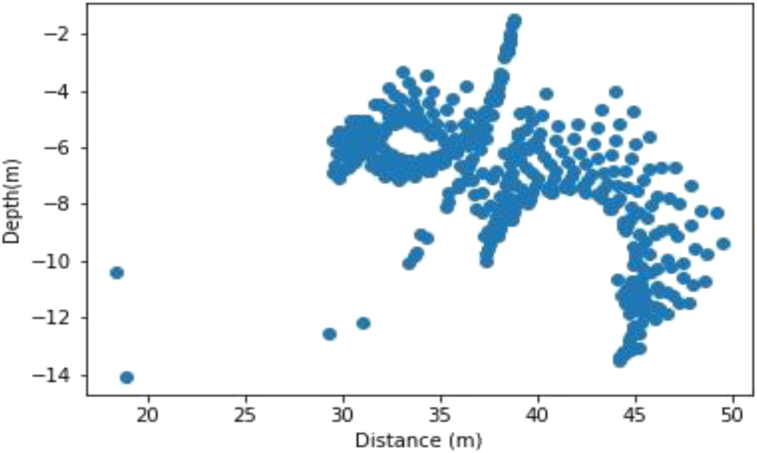
Initial plotting of the noise-free data before clustering (the blue color represents the solutions).

**Figure 5:**
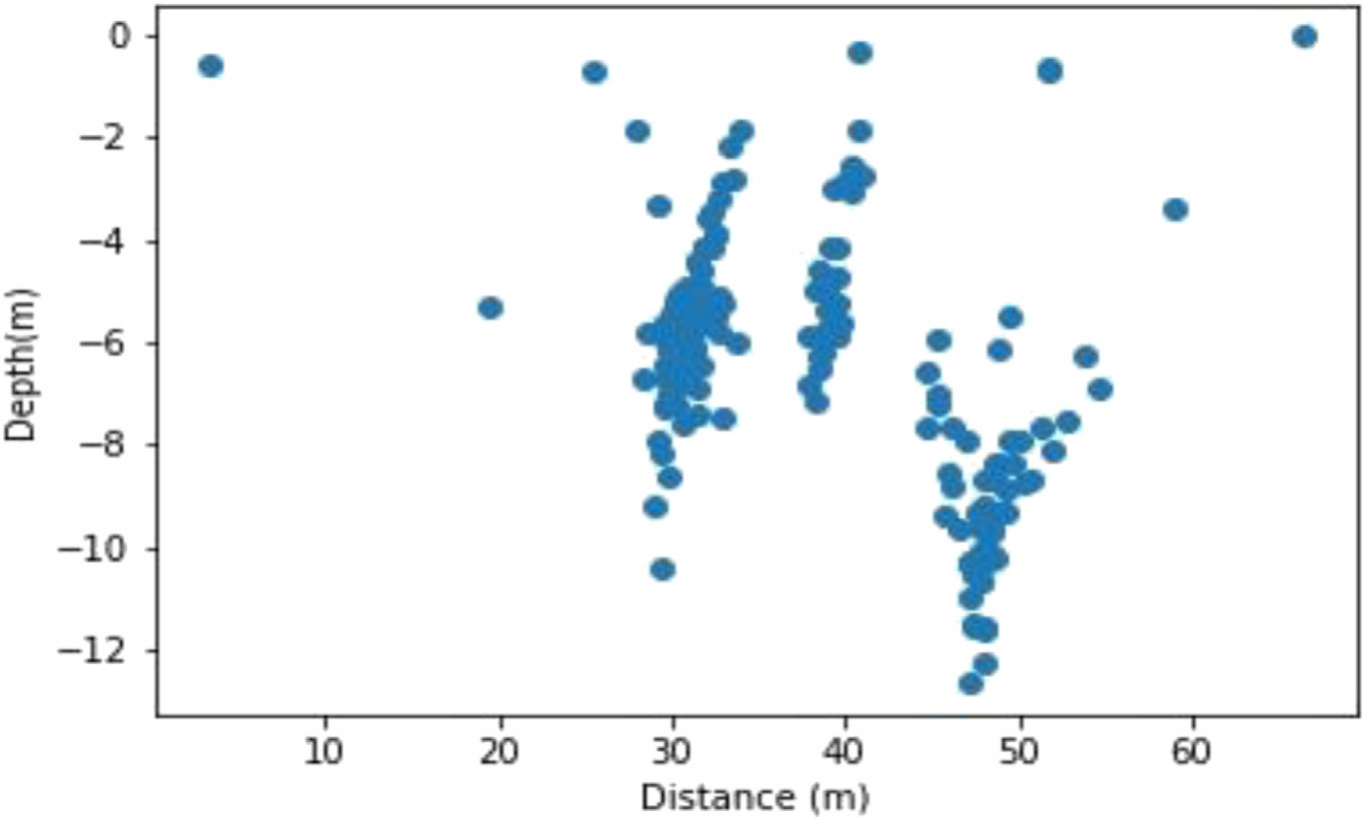
Initial plotting of the noise-added data before clustering (the blue color represents the solutions).

Figures 4 and 5 show the initial plot of the distance against depth, before applying the K-means clustering.

### 3.1 Modeling and analysis

Our computational strategy was to compute the results for all meaningful ranges of the input parameters (window width, interference polynomial, structural index). From the huge heap of results, we will pick up those that seem to be the best.

The K-means machine learning algorithm was applied to the two data sets. The major objective of the K-means is to group similar data points and discover similar patterns. The SciKit-learn library was used to import the necessary libraries for the K-means clustering implementation.

**Figure 6:**
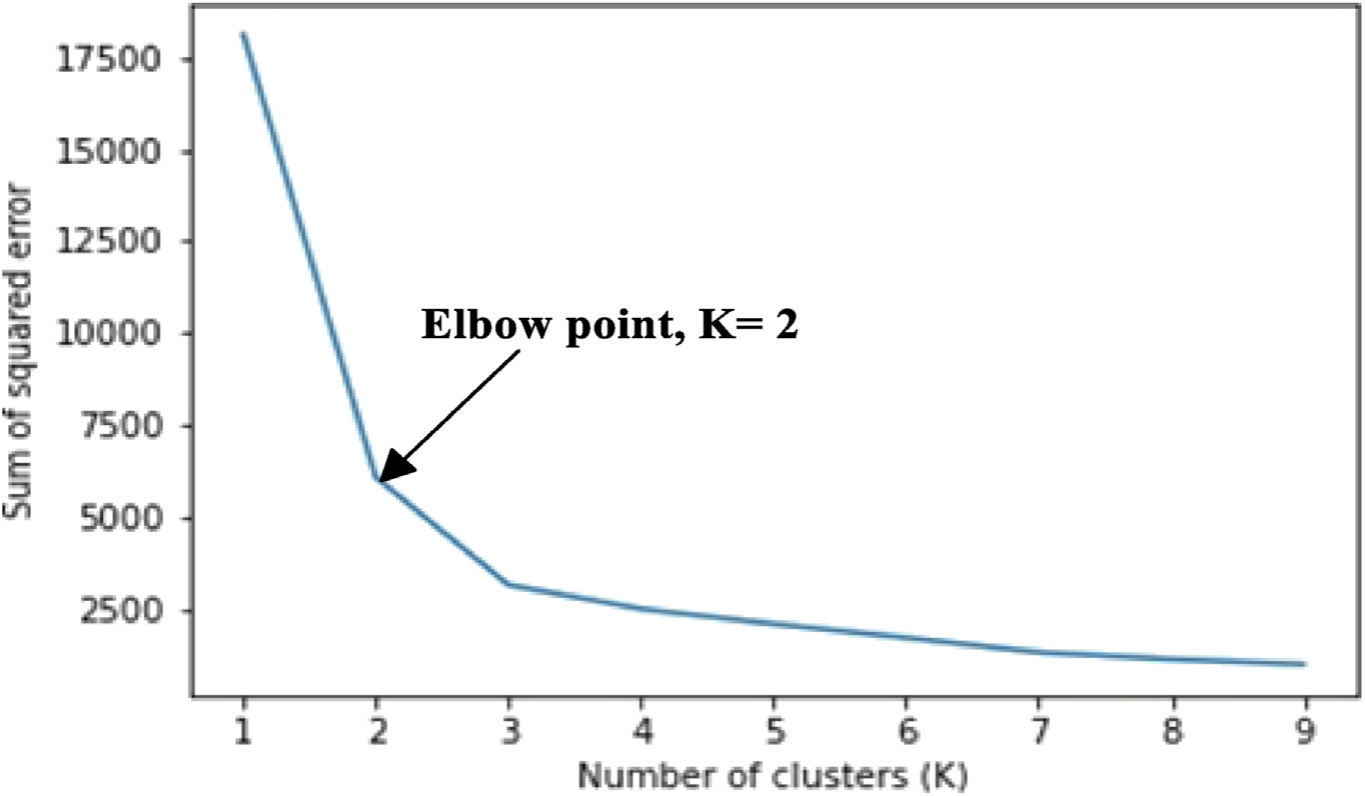
Elbow point for the noise-free dataset indicating that the optimum number of partition clusters is two (2).

**Figure 7:**
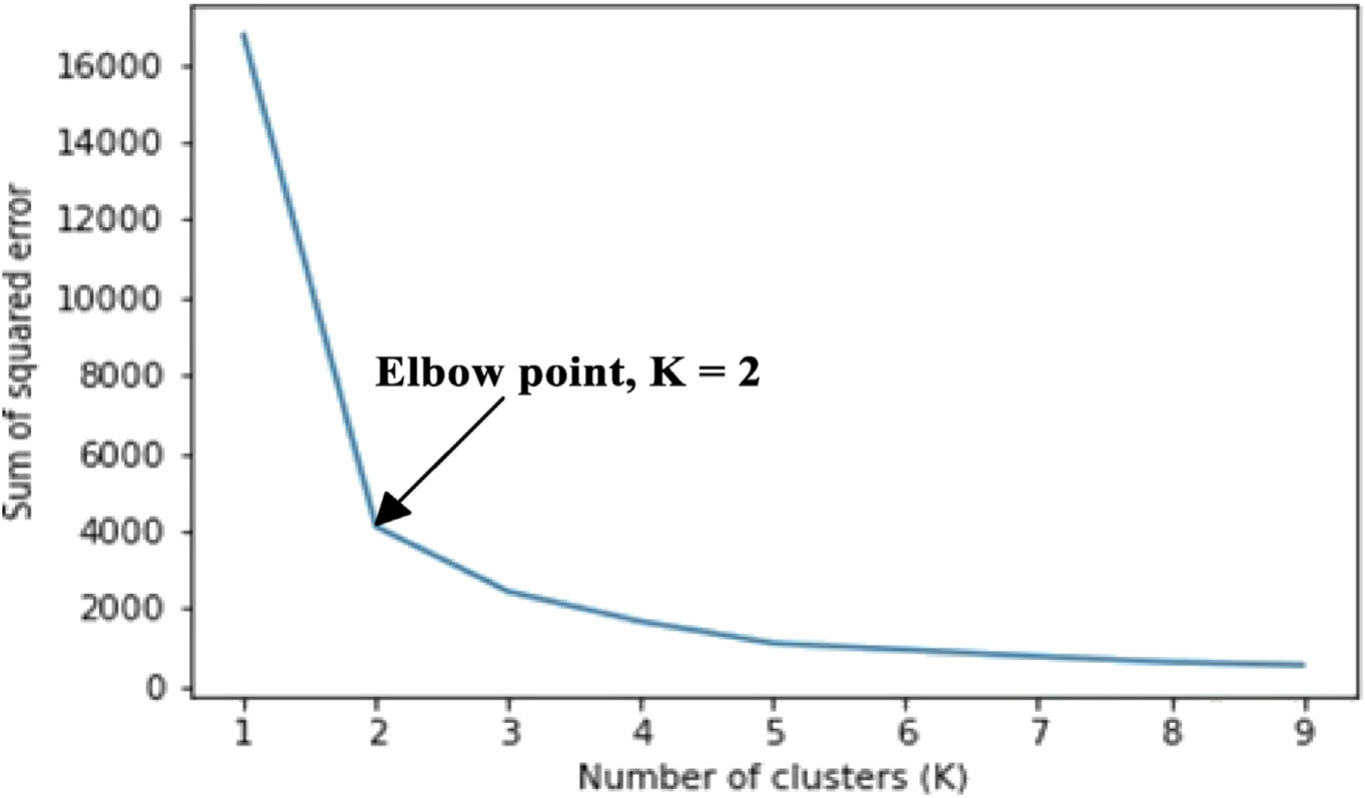
Elbow point for the noisy dataset indicating that the optimum number of partition clusters is two (2).

The elbow method was used first to determine the number of clusters before their centers were obtained using the K-means algorithm. Figures 6 and 7 show the number of clusters determined by the elbow method for the noise-free and noise-induced data. In this case, the K validity was found to be two (2).

The K-means algorithm was then applied to the generated solutions produced after Werner deconvolution was carried out on the synthetic model. This was done to see how effectively it can detect the number of dyke-like bodies that generated the solutions. K-means' main goal is to group similar data points and discover any similar patterns. K-means clustering will begin by locating k centers to form 'n' clusters. A cluster can be formed by aggregating similar data points based on specific functionalities and similarities. Centroids are imaginary or real locations at the center of a cluster identified by k-means clustering and forming k-centers. These k-centers connect the closest data points to the closest clusters, forming 'n' clusters while keeping the data centers as small as possible. The magnitude of the data (input and output) was scaled to ensure that the learning algorithm was not biased. After normalization, the data becomes stable within a given range of 0–1.

**Figure 8:**
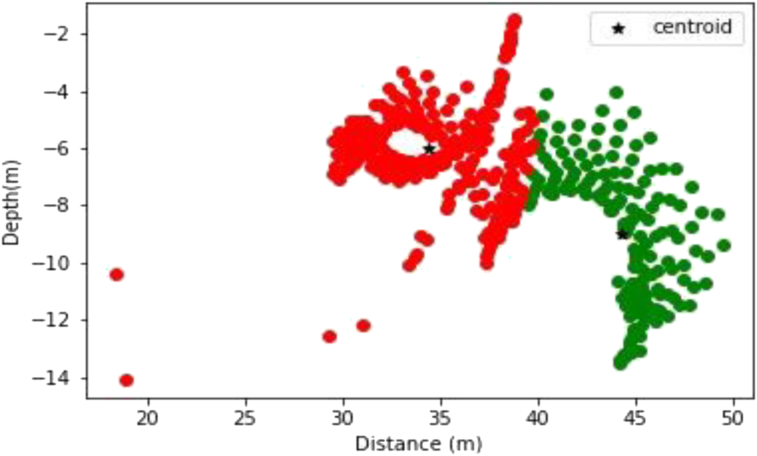
Visualization of K-Means cluster Analysis applied to the noise-free data (the green and red colors represent two partitioned clusters).

**Figure 9:**
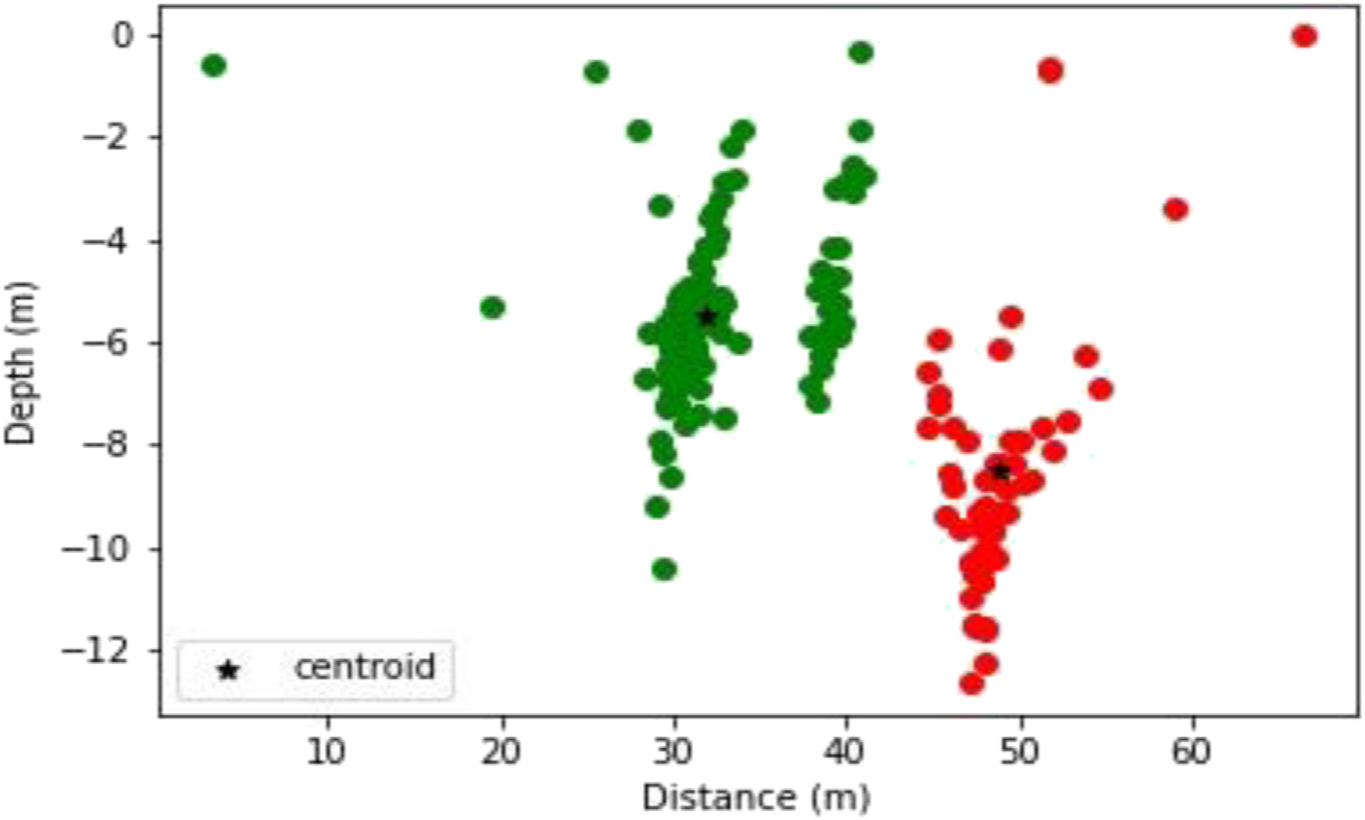
Visualization of K-means cluster analysis applied to the noisy data (the green and red colors rep two partitioned clusters).

For the noise-free data, the result of the K-means algorithm shows two clusters with two centroids (color red and blue) as shown in Figure 8. Figure 9 shows the k-means algorithm effect on the noise-enhanced data, with the two centroids also being shown.

**Figure 10:**
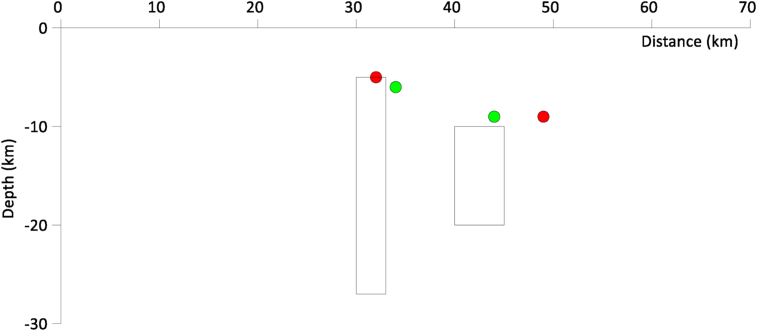
Visualization of K-Means centroids on the synthetic model (green and red color represents the centroid of both the non-noise and noisy data).

The centroids for both the noise-free and noisy data were plotted on the synthetic model, with the red and green dots representing the centroids for noise and noise-free data, respectively (Figure 10). It can be seen that the centers produced by the machine learning program closely approximate the depth to the top of the bodies in the model and the comparison with the true depths is shown in Table 2.

**Table:** 2Summary of the result showing the depth of the body before and after the K-means algorithm was applied.

Body	True model parameters		Solution using K-means (Noise-free data)		Solution using K-means (Noise added to data)	
	Depth to top (m)	Horizontal position (m)	Depth to top (m)	Horizontal position(m)	Depth to top (m)	Horizontal position (m)
A	5.0	31.5 (±1.5)	6 (20%)	34 (7.9%)	5 (0%)	32 (1.6%)
B	10.0	42.5 (±2.5)	9 (10%)	44 (3.5%)	9 (10%)	49 (15.3%)

Given that we just have two geologic dike shaped bodies from the magnetic synthetic model, we proceeded to perform Werner deconvolution to generate the solutions. K-means clustering algorithm was then applied to the solutions to reveal how effective the algorithm is in detecting the number of magnetic bodies, especially when noise was added. From the results, it was shown how successful the K-means algorithm could cluster magnetic solutions into two (2) clusters, revealing the true number of geologic bodies. After running the K-means algorithm a few times, it was found that the clusters were more or less stable. The centroids of each cluster were the position of the geologic bodies. For the less noise model, the values of the centroids for the two bodies were 6 m and 9 m in depth with a horizontal distance of 34 m and 44 m respectively. Considering the noise model, the values of the centroids were 5 m and 9 m in depth, at a horizontal distance of 32 m and 49 m respectively. This shows the effectiveness of the K-means algorithm in determining how many geological bodies created the solutions.

## 4 Application: Dangoma mining site, Kaduna, Nigeria

**Figure 11:**
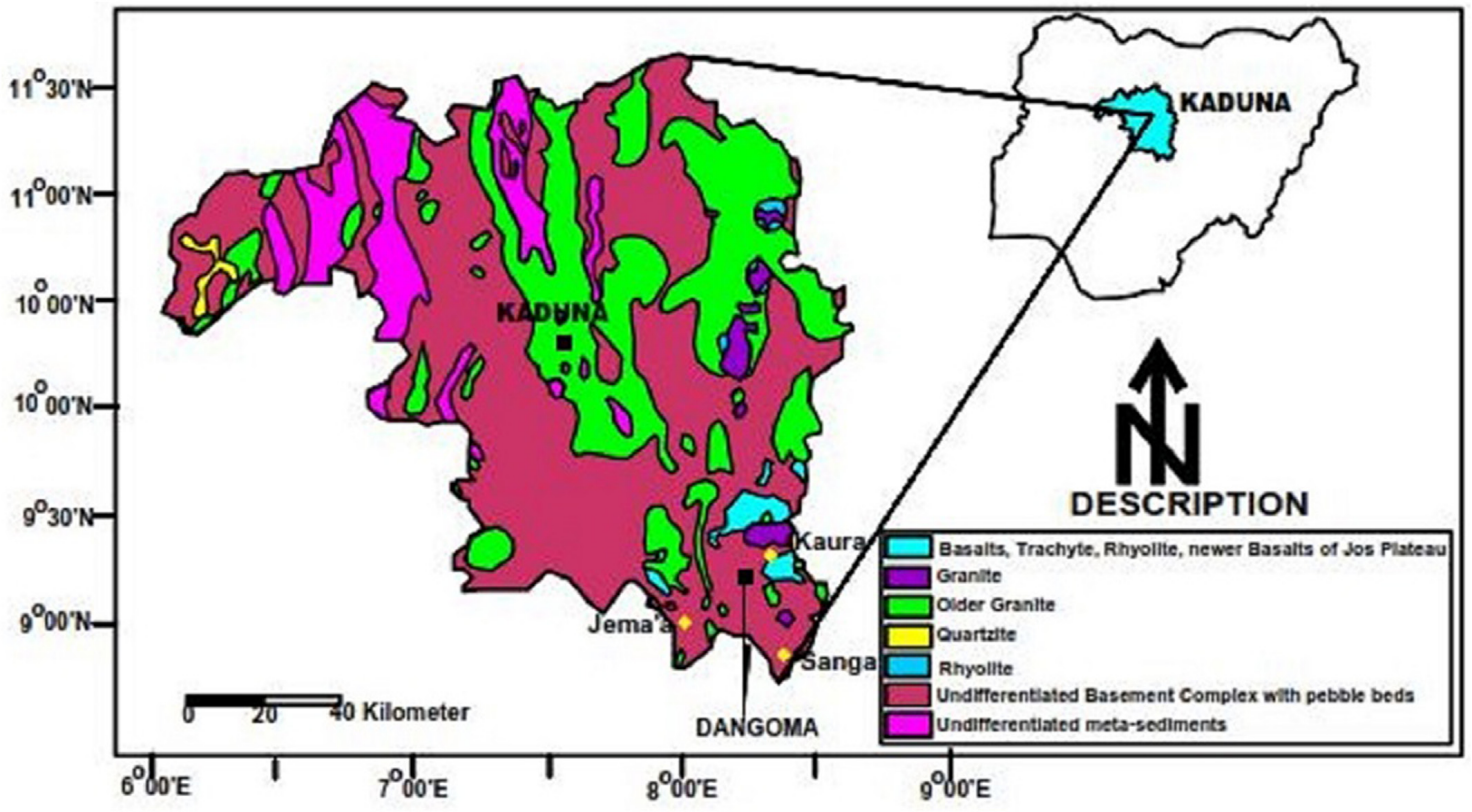
Location map of the Dangoma mining site, Kaduna, Nigeria (right) and regional geology (left).

Finally, we applied the algorithm to present a real data set at the Dangoma nickel deposit site in Kaduna, Nigeria. The site is one of the most potential nickel deposit districts in Nigeria. The area has an average elevation of about 700 m above sea level, is of low relief with slightly undulating topography and low-lying outcrops, and a few prominent ridges of weathered granite scattered over the landscape. The lateritic regolith is thick and deeply weathered. The Dangoma area is primarily the Precambrian Basement Complex which is composed of the mostly Archean migmatite-gneiss complex, Proterozoic schist belts, and the Pan-African Older Granites intruded by minor meta-ultramafic bodies and pegmatite dykes (Oyawoye, 1970; Ajibade and Wright, 1988). The Dangoma area lies east of the N–S trending supracrustal schist belts of northwestern Nigeria (Figure 11), it is underlain by migmatites, gneisses, (serpentinite), and granites. An airborne geophysical survey was ﬂown in 2006 to aid in the geologic mapping and exploration of Nigeria. The magnetic sensor was towed at an altitude of 80 m, with a line spacing of 200 m. In each of the four subblocks into which the land was divided, the flight lines were parallel to the principal geologic strike.

**Figure 12:**
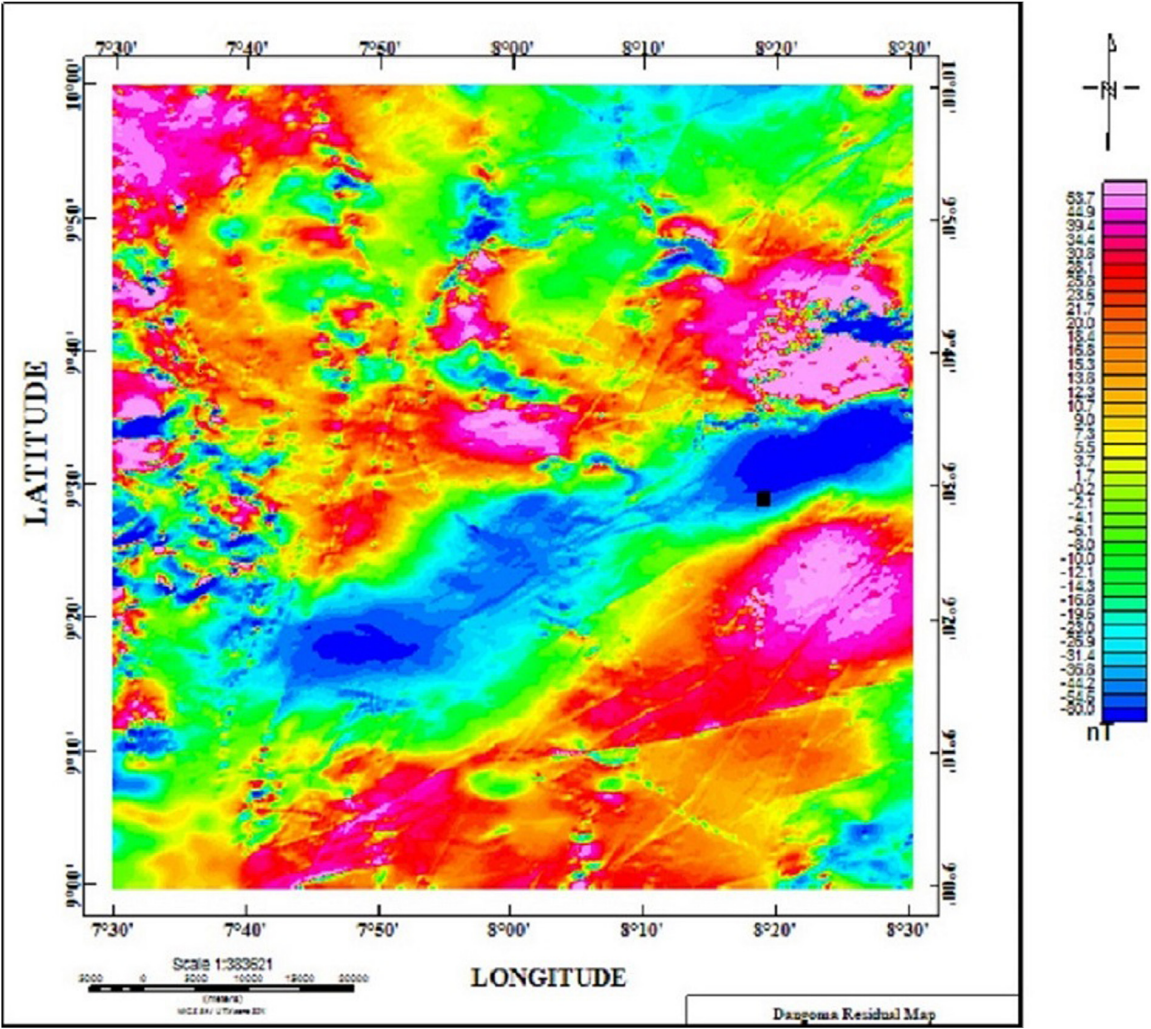
Residual magnetic intensity map over the study area (the black box shows the location of the deposits).

Figure 12 displays the Residual Magnetic Intensity data over a window of the region where we evaluated the clustering algorithm and Werner deconvolution The magnetic data show the contrast between weakly magnetic felsic volcanic units and basalts, gabbros, and strongly magnetic pegmatite dike and intrusive units. The location of the mineral deposit in the area is shown by the black box on the Residual map, for reference. The correlation between the data sets is good, although some areas apparently do not have any associated magnetic signature, like the folded units on the southeast part of the map.

As with the synthetic data set, the Werner deconvolution was applied across a profile drawn on the Residual Magnetic data. The algorithm produced approximately 200 solutions with depths between 0 and 2400 m a window size of 100 m was used. A magnetic susceptibility value of 0.002549 SI was recorded at a dip angle 48.1°, and this occur at the distance of 38000m along the profile, with depth values ranging from 500 m to 2400 m.

**Figure 13:**
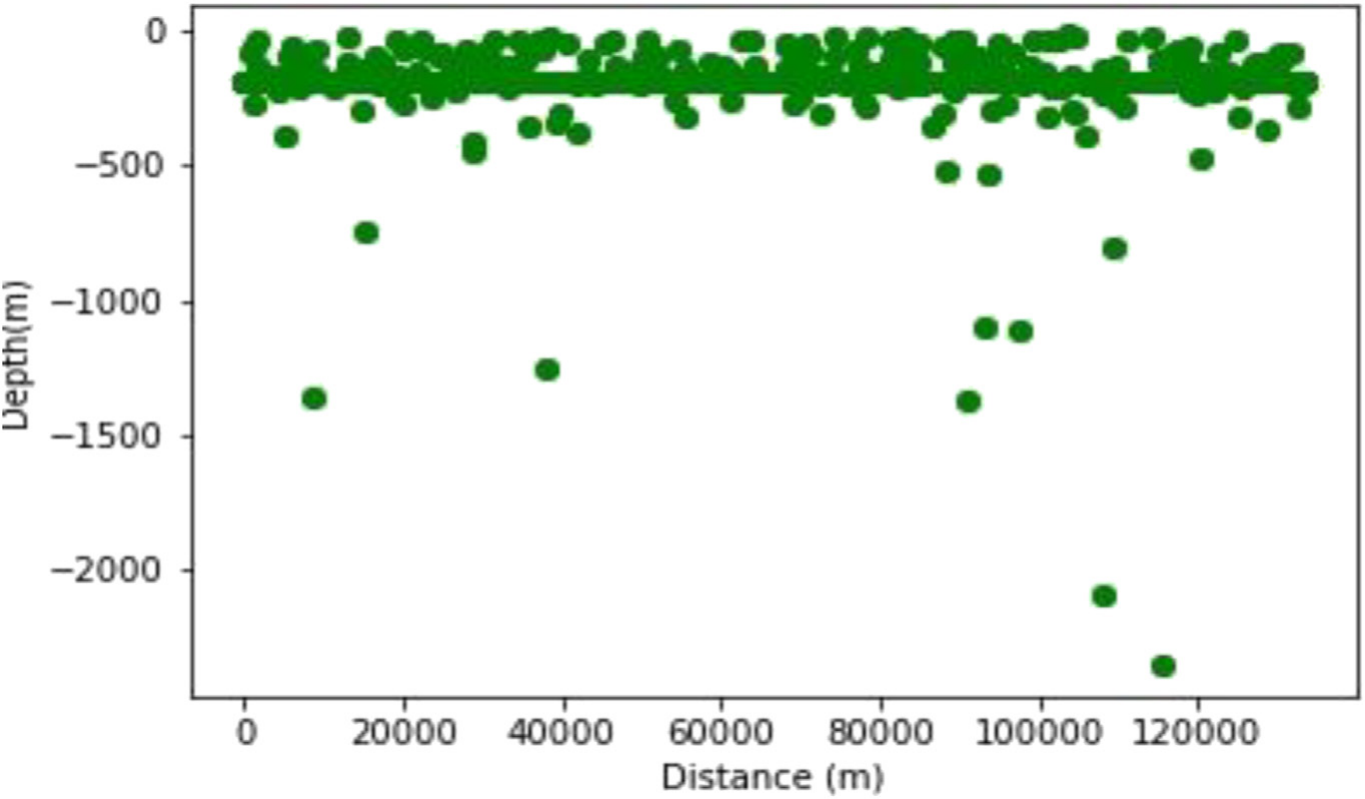
Initial plotting of the Werner solutions on the Dangoma data with susceptibility 0.002549 SI set before the application of the K-means algorithm.

The generated Werner solutions are as shown in Figure 13. The elbow method was then used to determine the number of partition clusters before their centers were obtained using the K-means algorithm.

**Figure 14:**
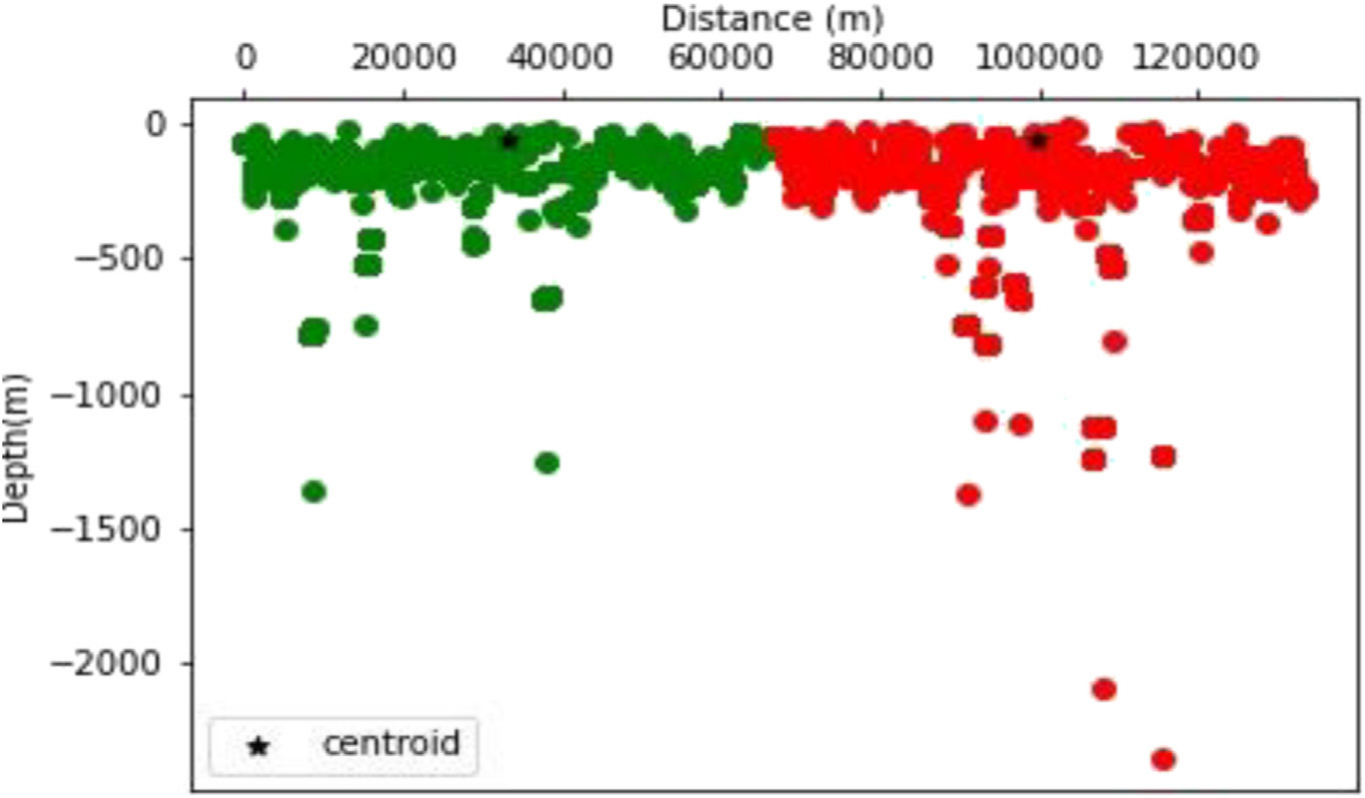
Visualization of K-Means cluster Analysis applied to the Dangoma data set with susceptibility of 0.002549 SI (the green and red colors represents two partitioned clusters).

The K-means clustering technique was applied to this data set. After experimenting with various cluster numbers and determining the best, an ideal number of two clusters was determined. The algorithm's strike information closely matches the folded structures in the Dangoma area, and cluster locations are depicted in Figure 14. This data should be useful for any future geophysical modeling efforts in the region.

## 5 Discussion

This method is far from foolproof for structural mapping from geophysical data. A wise selection of the various parameters required for the computation is critical to obtaining good results. The results of the clustering algorithm on a high-quality Werner data set, on the other hand, are extremely reasonable and should be useful as input for structural analysis and geologic modeling.

The Werner deconvolution technique is a broadly applicable technique for the analysis of 2-D potential field data. The ease with which it is programmed on a digital computer allows for quick analyses of reconnaissance aeromagnetic and gravity data, and its automatic regional-residual separation. These are its two principal uses. Kitty (1983) showed that its main flaws are sensitivity to geologic noise, field data noise, and the relatively high correlation between the metrics defining interference and those describing the desirable features of the anomaly-causing entity. This served as the bases for applying the K-means machine learning algorithm.

In general, unless one considers that there is no interference, the effect of this correlation is to only provide a certain amount of information about the depth and location of the causative body. Werner deconvolution is less preferable than forward or inverse modeling when more in-depth information is needed due to the amount of scatter in the position and depth estimates. Without a doubt, the Werner deconvolution's chosen algorithm makes or breaks the end product (geological imaging and depth estimations). The data will be filtered using the methods described here even if the Werner solution data set is not properly confined; nevertheless, the strike and depth information gained will only be useful if the data are closely connected to one another and follow real geologic structures. The Werner solutions utilized here were subjected to the machine learning technique, which produced good results in terms of solution coherency; as a result, the strike information in both the synthetic and actual data examples was accurate. Although the methodology was applied mostly to derive the geologic and depth of anomalous bodies, it undoubtedly can be applied to other problems such as model construction.

The algorithm showed the ability to detect a number of geologic bodies that produced the solutions. These tool has provided alternative ways of learning from data, and lend themselves naturally to different data types, with machine learning being well suited to high-dimensional data. K-means is a good first choice of algorithm that can be applied for geological clustering Importantly, the K-means machine learning algorithm is relatively straightforward to implement by a non-machine learning expert. Using machine learning before geophysical inversion allows information from a greater variety of data sources to be used to improve detail in 2D Werner deconvolution mapping.

The K-means clustering algorithm is very stable. Both applications (synthetic and real data) gave very good results in terms of partitioning the data into proper clusters and recognizing the major body generating the solutions.

## 6 Conclusion

In the presented contribution we analyzed solutions for 2D semi-automated inversion methods, namely Werner deconvolution to improve the results. For this purpose, the K-mean machine learning algorithm was applied to the Werner solutions. When good choices are made for the k-mean clustering parameter, it is a useful machine learning tool that can cluster Werner solutions and predict the number of geologic bodies that produced such solutions. This will enhance how subsurface structures are characterized, especially in places of complex geology where human intelligence may be limited.

The algorithm was tested on one synthetic data set and one real data set at the Dagogoma mining site in Kaduna, Nigeria. By using the Werner-generated solutions as input, the K-means algorithm was able to separate the clusters at the mining site and pinpoint the general location of the geologic formations. The best solution set must be found by running the K-means clustering algorithm with various cluster sizes. For large data sets, this is computationally inefficient and can be very slow. Further research is also required in the domain of curvature-based magnetic source filtering to reduce anomalous interference effects prior to Werner deconvolution.

## Declarations

### Author contribution statement

Daniel Eshimiakhe: Conceived and designed the experiments; Performed the experiments; Analyzed and interpreted the data; Contributed reagents, materials, analysis tools or data; Wrote the paper.

Kola Lawal: Conceived and designed the experiments; Contributed reagents, materials, analysis tools or data.

### Funding statement

This research did not receive any specific grant from funding agencies in the public, commercial, or not-for-profit sectors.

### Data availability statement

Data will be made available on request.

### Declaration of interests statement

The authors declare no conflict of interest.

### Additional information

No additional information is available for this paper.
